# A Case of Purpura Hemorrhagica Successfully Treated

**Published:** 1840-06

**Authors:** William W. Searcy

**Affiliations:** Nashville, Tennessee


Art. IV.—*A Case of Purpura Hemorrhagica successfully treated.* By Wm. W. Searcy, M. D., of Nashville, Tennessee. Reported to the Medical Society, of Tennessee, at its 11th annual meeting.

On the 18th of August last, I was requested to visit a young girl, aged 12 years, who, I was informed, had been laboring under an attack of fever of nine days duration, but had been convalescing. The situation of the girl when I first saw her was as follows:—she was very pale, and could not be raised for fainting; her surface was unnaturally cool; great restlessness; some thirst and headache; tongue pale and flabby; pulse barely perceptible and extremely frequent; *much tenderness* of epigastrium to pressure; her bowels constipated. There was a constant oozing of blood from the nares and throat, which had existed for two days and nights uninterruptedly, which caused extreme exhaustion. There was discovered about the face a few red and blue blotches, and a further examination revealed a numerous eruption of a similar kind on the body, limbs, insides of the lips and cheeks. They varied from the size of a pin head to a four-penny piece.

The girl has always been rather delicate, of a nervous, sanguine temperament. She has never had a similar eruption; bleeds frequently from the nose when in health, but never before to an alarming or exhausting extent. Her flesh when wounded is not disposed to bleed more profusely than similar wounds in other persons; none of her relations have been known to have a similar eruption or to be subject to profuse hemorrhages. The scrofulous diathesis is not known in the family.

Four grains of acetate of lead were ordered to be administered every half hour until the hemorrhage should cease; also a strong solution of the same (1 drachm to a pint of water,) to be repeatedly injected up the nares. A blister was applied to the nape of the neck and one to the calf of each leg. Diet, water gruel, chicken broth, or rice water; drink, lemonade.

The next morning, 19th, I was informed that the hemorrhage had ceased after the seventh dose of lead, and had not again returned; the blisters had drawn well. Her extremities and surface at present are much warmer; pulse not so feeble, but still very frequent; the patient is very restless, has slept but little for two or three nights; complains very much of her blisters, has but little thirst and no appetite; *great tenderness* of the epigastrium to pressure; and bowels constipated. The last passage from her bowels was dark and liquid. Her tongue is pale and flabby; there is a pretty numerous new crop of petechiæ; those of yesterday which were small and red, have enlarged and are of a bluish cast. A mercurial purge of 12 grains of calomel was ordered, and every two hours 6 drops of elixir vitriol and 1 grain of sulph. quinine. Diet and drink continued.

20th. Patient's condition not materially altered since yesterday; calomel has operated but once, the evacuation copious, dark, and liquid. She had slight fever yesterday evening. There is still much tenderness of epigastrium; pulse and tongue vary but little. Ordered castor oil, and a continuation of vitriol and quinine, and the same diet and drink.

21st. After taking oil yesterday, patient's bowels were freely purged; discharges more consistent and yellow; rested very well and slept soundly all night; her appearance and symptoms every way much more favorable; she feels much stronger and desires to eat; very few fresh petechiæ, and old ones fading considerably; tenderness of epigastrium much less. Ordered a continuance of quinine and vitriol, and diet a little more nourishing.

22d. This morning patient says she does not feel so well as on yesterday; indulged rather freely in eating, and had some fever in the evening; rested badly last night; eruption fading away and no new specks discovered; pulse frequent but feeble; headache; bowels have not been moved since yesterday morning; epigastrium *tender* again to pressure; there has been no return of the hemorrhage. Ordered castor oil; continue quinine and vitriol, and restrict her diet exclusively to water gruel, chicken broth, or rice water, and use acid drinks. Visited her this evening, found her with some fever and headache. Oil which she took this morning had operated but once; prescribed an additional dose of oil; discontinue quinine and vitriol and use citrated kali during pyrexia.

23d. Patient expresses herself much better this morning; had three copious discharges early last evening, consistent and yellow; tenderness of epigastrium very much diminished; fever left her early in the evening, has rested well all night; free from headache; feels much stronger and desires to eat. Temperature of the surface and extremities more natural; tongue looks well; pulse fuller, soft and not so frequent. The eruption is now barely visible. Ordered castor oil and resume quinine and vitriol.

24th. Patient convalescing—omit the tonic and take oil enough to keep the bowels moderately soluble.

25th and 26th. Patient still convalescing and continued from this out to mend rapidly. Up to this date, 4th May, 1840, has had no return of the disease and her health is excellent.

*Nashville*, May, 4, 1840.

